# Allicin and Cancer Hallmarks

**DOI:** 10.3390/molecules29061320

**Published:** 2024-03-15

**Authors:** Wamidh H. Talib, Media Mohammed Baban, Aya O. Azzam, Jenan J. Issa, Alaa Y. Ali, Alia Kh. AlSuwais, Sana Allala, Lina T. AL Kury

**Affiliations:** 1 Faculty of Allied Medical Sciences, Applied Science Private University, Amman 11931, Jordan; 2 Department of Clinical Pharmacy and Therapeutics, Applied Science University, Amman 11931, Jordan; mediababan22@gmail.com (M.M.B.); ay_azzam@asu.edu.jo (A.O.A.); jenanjamal@hotmail.com (J.J.I.); aliakh.alsuwais@outlook.com (A.K.A.); sanaallala8@gmail.com (S.A.); 3 Department of Health Sciences, College of Natural and Health Sciences, Zayed University, Abu Dhabi 144534, United Arab Emirates

**Keywords:** allicin, cancer hallmarks, anticancer, angiogenesis, metastasis, immune evasion

## Abstract

Natural products, particularly medicinal plants, are crucial in combating cancer and aiding in the discovery and development of new therapeutic agents owing to their biologically active compounds. They offer a promising avenue for developing effective anticancer medications because of their low toxicity, diverse chemical structures, and ability to target various cancers. Allicin is one of the main ingredients in garlic (*Allium sativum* L.). It is a bioactive sulfur compound maintained in various plant sections in a precursor state. Numerous studies have documented the positive health benefits of this natural compound on many chronic conditions, including gastric, hepatic, breast, lung, cervical, prostate, and colon cancer. Moreover, allicin may target several cancer hallmarks or fundamental biological traits and functions that influence cancer development and spread. Cancer hallmarks include sustained proliferation, evasion of growth suppressors, metastasis, replicative immortality, angiogenesis, resistance to cell death, altered cellular energetics, and immune evasion. The findings of this review should provide researchers and medical professionals with a solid basis to support fundamental and clinical investigations of allicin as a prospective anticancer drug. This review outlines the anticancer role of allicin in each hallmark of cancer.

## 1. Introduction

**Figure 1:**
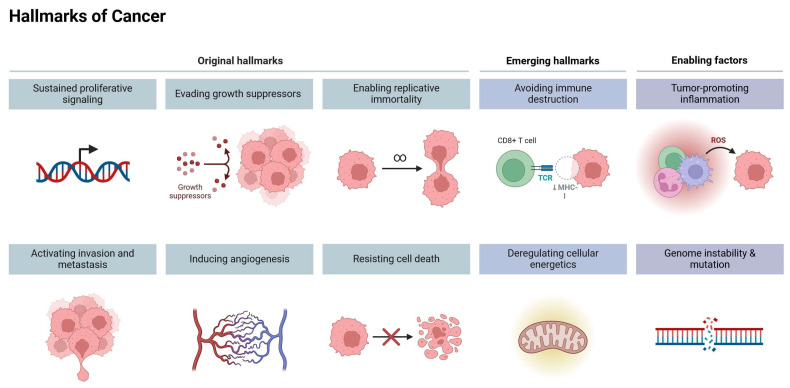
Summary of the potential cancer hallmark targets for allicin.

Cancer, the second-leading cause of death worldwide, is one of the biggest public health problems, resulting in the annual mortality of 10 million people [1]. Because of the detrimental effects that cancer and its treatment have on financial resources and the healthcare system, greater focus must be paid to creating novel preventative and therapeutic approaches that are both affordable and efficacious [2]. Natural products are a viable source for the development of new, effective anticancer medications because of their low toxicity, diversity of chemical structures, and capacity to target many cancers [3]. One of these compounds is allicin which is a potent bioactive in garlic [4]. Its antitumor potential is reported in a variety of tumor types [5]. Furthermore, antibacterial [6], cholesterol-lowering [7], anti-inflammatory [8], and antiviral activities of allicin are also described [9]. Several studies have stated that the majority of the lipid-lowering, antioxidant, anti-atherosclerotic, and anticancer effects of whole garlic, as seen in animals and humans, are submitted to allicin, or their spontaneous transformation compounds (allyl polysulfides), or their common metabolite (allyl methyl sulfide, AMS) [10,11]. Interestingly, an increasing amount of research indicates that allicin may target several cancer hallmarks, which are the basic biological functions and characteristics that contribute to the occurrence and progression of cancer [12]. The cancer hallmarks include sustained proliferative signaling, evasion of growth suppressors, resistance to cell death, replicative immortality, induction of angiogenesis, activation of invasion and metastasis, reprogramming of energy metabolism, and evasion of immune destruction [13]. It has been reported that allicin inhibits cancer cell proliferation, induces cell apoptosis, and enhances the accumulation of reactive oxygen species [14,15,16]. Figure 1 shows a summary of cancer hallmarks that can be potentially targeted by allicin.

This review summarizes the antitumor activity of allicin against various cancers through each of the eight cancer hallmarks. It is hoped that this review will provide essential knowledge regarding cancer treatment by allicin.

## 2. Allicin

### 2.1. Allicin Chemical Structure and Formation

**Figure 2:**
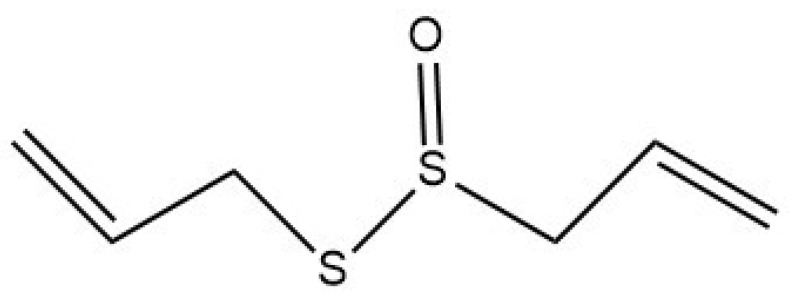
Allicin chemical structure [S-(Prop-2-en-1-yl) prop-2-ene-1-sulfinothioate].

Garlic (*Allium sativum* L.) contains many well-known organosulfur compounds, including allicin or diallyl thiosulfinate (Figure 2). Garlic does not naturally contain allicin; it can only be formed when the cloves are sliced or crushed [17]. Freshly crushed garlic has a distinct odor due to this volatile molecule (allicin), which is weakly miscible in water [18]. When the garlic clove is chopped or cleaved, it activates the alliinase enzyme. Following that, this enzyme transforms the amino acid alliin (L-(+)-S-Allyl cysteine sulfoxide) into allyl sulfenic acid (2-propene-sulfenic acid), which is unstable and highly reactive at room temperature. Then, two allyl sulfenic acid molecules spontaneously condense to generate allicin [19]. Diallyl sulfide (DAS), diallyl disulfide (DADS), diallyl trisulfide, allyl methyl trisulfide, dithiins, and ajone vinyldithiines are some of the metabolites of allicin [20].

### 2.2. Allicin Bioavailability

Despite many studies on the biological effects of garlic’s sulfur compounds, we know little about how those compounds are metabolized and their associated bioavailability [21]. Clinical trial data on garlic’s anticancer properties are inconsistent due to variations in the bioavailability of sulfur-containing components between raw garlic and supplement formulations containing allicin as the main pharmacological agent [11]. Studies revealed that garlic extract possesses growth-inhibitory features that are attributed to its water-soluble component, allicin [22]. It is produced by an enzyme alliinase, which reacts to an allin precursor when garlic is cut or chewed [23]. Fortunately, the half-life of allicin has been proven to be less than a minute, which indicates that it is digested quickly [24] due to its quick penetration into several cell compartments. Allicin is highly unstable and thus has strong membrane permeability and significant antioxidant activity [17]. It can be stabilized by monoclonal antibody conjugation and liposome encapsulation; this may also help in reducing its unpleasant aroma. Besides the above-mentioned pharmacological properties, allicin also exhibits high gastrointestinal absorption and good blood–brain barrier permeability [25,26]. Several methods are considered to enhance the bioavailability and anticancer effects of allicin, such as nanoformulations. For instance, a study suggested the use of cyclodextrin-based nanoparticles to improve the cellular delivery of allicin. This approach significantly enhanced the efficacy of allicin in cancer treatment [27]. Recently, researchers utilized this method and encapsulated the compound in solid lipid nanoparticles (SLNs) coated with chitosan-conjugated folic acid and administered it. The results revealed that these nanoparticles have the maximized potential to trigger apoptosis and prevent free radicals in cancer cells by stimulating the intrinsic apoptosis pathways [28]. Remarkably, another study prepared allicin nanoformulations using gelatin nanoparticles (GNPs) surface-conjugated to glycyrrhetinic acid. The application of these particles resulted in increased allicin cytotoxicity towards liver cancer cells (HepG2), suggesting them a successful liver cancer therapy [29]. In addition, an allicin-loaded folic acid and polyethylene glycol (PEG)-modified chitosan/lecithin formulation was also developed to enhance the anti-colon cancer effects of allicin. Their application notably increased allicin toxicity by upsurging caspase 3 and 9 expression and activation of intrinsic apoptotic cascade in treated cancer cells. Additionally, it also confirmed allicin’s anti-angiogenic effects [30]. Concludingly, combining allicin with several nano-carriers may not only increase its cellular delivery and bioavailability but also have therapeutic implications.

### 2.3. Allicin Toxicity

A randomized controlled trial has shown that high doses of allicin in susceptible individuals can result in a range of side effects such as insomnia, vomiting, heartburn, dizziness, diarrhea, tachycardia, sweating, offensive body odor, and flatulence [31]. Chemically, allicin is a lipid soluble so it can cross the cell membrane and enter the cells easily to oxidize cellular thiols and result in structural protein changes [4]. A meta-analysis including eight studies of garlic plants showed that the increased intake of allium vegetables was associated with an increased risk of colon disease in women and using garlic supplements can increase the chances of colorectal cancer (CRC) [32].

### 2.4. Biological Functions

Allicin and its secondary metabolites have many important biological functions, such as anticancer effects. It not only protects against tumors but also alleviates the adverse effects of anticancer treatment and enhances the chemotherapeutic response [19]. Allicin has an antioxidant, anti-inflammatory, antihypertensive, and cardiovascular protective role in our bodies [33]. As an antioxidant phytochemical, it scavenges reactive oxygen species (ROS) and protects cells from oxidative DNA damage [34]. Alongside this, allicin exhibits telomerase activity inhibition in both dose- and time-dependent manners in gastric cancer cells. Thus, it can serve as a potent anticancer agent [25]. Moving forward, several studies have indicated that it potentially affects cancer cell growth, proliferation, and metastasis and encourages apoptosis (programmed cell death) [4,35]. Allicin also has an antipathogenic effect against bacteria, viruses, fungi, and parasites. On the other hand, it can increase the gut’s normal flora (beneficial bacteria) [4].

## 3. Cancer Hallmarks as Therapeutic Targets

Carcinogenesis is a complex, coordinated process that progresses from normal cells to metastatic disease. To better understand this process, Hahn and Weinberg [36] proposed six hallmarks. In 2011, the same scientists increased the number of these hallmarks to eight [37], which include sustained proliferation, evading growth suppressors, metastasis, replicative immortality, angiogenesis, resisting cell death, altered cellular energetics, and immune evasion [38]. It is now feasible to suppress tumor development, trigger cell death, and stop the metastatic spread by developing medicines that specifically disrupt these hallmarks, such as inhibitors of oncogenic signaling, anti-angiogenic agents, or medications targeting important molecules involved in invasion or metabolism [38]. The continuous development of targeted therapies holds promise for improving treatment outcomes and reducing the adverse effects of conventional non-specific cancer cures [39]. However, to fully comprehend the complex mechanisms of cancer development and malignant progression, it is crucial to explore and expand the original hallmarks of illnesses. Douglas Hanahan introduces potential additions to the conceptualization of the hallmarks of cancer, such as cellular plasticity, epigenetic changes, microorganism influence, and neuronal signaling impact [38]. Herein, the potential of this knowledge can be utilized in the future to create novel target therapeutic strategies for proactively managing cancer progression [40]. Nevertheless, understanding cancer hallmarks may provide effective therapeutic targets but not disease prevention [13].

## 4. Allicin and Cancer Hallmarks

### 4.1. Role of Allicin in Genomic Instability

Genomic Instability is a crucial characteristic of all cancer cells [41]. It primarily manifests as chromosomal, intrachromosomal, microsatellite, and epigenetic instability and has been associated with malignant cancers [42]. The genome’s integrity is protected by checkpoints in normal cells, while in cancer, aneuploidy or an abnormal number of chromosomes leads to the failure of these checkpoints [43]. These checkpoints are regulated by proteins that either promote or prevent cell division, which are typically altered in cancer cells to stimulate uncontrolled growth [44]. DNA replication, repair processes, and cell-cycle checkpoints all contribute to the maintenance of a cell’s genome’s integrity [45]. Although genomic instability promotes pathological conditions, including cancer, hereditary illnesses, and premature aging, it also helps species diversification, immunoglobulin diversification, and evolutionarily conserved checkpoints, which are essential [46]. Moreover, the accumulation of genetic and epigenetic changes in healthy cells may upsurge mutation rates or increase genomic instability, which raises the risk of cancer development [47]. To reduce genomic instability, five strategies were suggested: minimizing DNA damage, promoting DNA repair, focusing on DNA repair issues, addressing centrosome clustering issues, and suppressing telomerase activity [48]. Nuclear factor erythroid 2-related factor 2 (NRF2), which controls the antioxidant system in cervical epithelial tissue, preserves homeostasis by preventing the transformation of normal cells and promoting the proliferation and survival of cancer cells. An interesting study revealed that (40 nM) allicin inhibited the expression of NRF2 in cervical cancer SiHa cells, thus illustrating its potential therapeutic advantages in cervical cancer treatment [49]. Moreover, telomerase is a crucial enzyme in cell division, facilitating telomere elongation and DNA replication, which is vital for cancer cells [50]. Allicin specifically suppresses telomerase activity, which lowers the growth of cancer cells in a dose-dependent way [51]. In accordance, allicin at concentrations of 0.016 mg/mL, 0.05 mg/mL, and 0.1 mg/mL effectively suppresses telomerase activity and increases apoptosis in gastric adenocarcinoma cells (SGC-7901) [52].

### 4.2. Induction of Apoptosis

Organosulfur compounds (OSCs) have been demonstrated to have anti-inflammatory, antioxidant, and anticancerous properties [53]. In vivo studies have established that OSCs can reduce the incidence of colon cancer by causing mitotic arrest and apoptosis [54,55]. The potential of allicin to cause apoptosis was considered to be the cause of its anticancer effects [56]. Apoptosis can occur through various pathways, including both caspase-dependent [5] and caspase-independent pathways [57]. Apoptosis-inducing factor (AIF), the first mitochondrial-released protein, regulates caspase-independent apoptosis and promotes chromatin condensation and DNA deterioration [58]. Interestingly, in human colon cancer HCT-116 cells, Chen et al. [59] showed that allicin induces Nrf2, raises hypodiploid DNA content, raises Bax levels, and lowers B-cell non-Hodgkin lymphoma-2(Bcl-2) levels, which together promote apoptosis. Thus, organosulfur compounds (OSCs) or allicin can act as effective tumor suppressor agents.

#### 4.2.1. Caspase-Dependent Apoptosis

Apoptosis is initiated by signals from both intracellular and extracellular compartments, either through the extrinsic/extracellular pathway or the intrinsic pathway, which are caspase-dependent [60]. Oxidative stress causes the induction of pro-apoptotic proteins (cyt c) from the mitochondria, which leads to the activation of caspase-9 and caspase-3 proteins, eventually resulting in apoptosis [61]. A study by Rosas-González et al. showed that allicin decreased cell viability and induced apoptosis pathways in breast cancer cell lines luminal A (MCF-7 cells ) treated with 45 μM allicin and in triple-negative (HCC-70) cells treated with 12 μM, 20 μM, and 45 μM concentrations [62]. Moreover, allicin 30 and 60 µg/mL has a potential anticancer effect against Glioblastoma (GBM), since it slows the growth and causes apoptosis in glioma cells in vitro by activating both intrinsic mitochondrial and extrinsic Fas/FasL-mediated pathways [63]. Regarding apoptosis resistance, the combination of allicin (10 µM) and Cisplatin (10 µM) causes autophagy-dependent cell death in SW1736 and HTh-7 cells, indicating that allicin may be used in conjunction with other therapies to treat thyroid cancer [64]. Moreover, the capacity of allicin to decrease NRF2 is what essentially causes it to promote cell apoptosis in cervical cancer cells with (40 nM) [49]. Further analysis showed that allicin (40 μg/mL) causes G2-M arrest and death in A549 cells in Non-Small Cell Lung Cancer (NSCLC) through ROS-dependent changes to p53, p21, and other downstream effectors in vitro [65]. According to in vivo research, allicin 10 and 20 mg/kg was administered, i.e., daily for 4 weeks, and it increased the apoptotic caspases that were produced in Cholangiocarcinoma (CCA) by suppressing STAT3 signalings, such as cleaved caspase-3 and cleaved caspase-9 [66].

Another research study reported that treatment of murine T-lymphocytes (EL-4) cells with 1 µg/mL, 4 µg/mL, and 8 µg/mL concentrations of allicin for 24 to 60 h significantly inhibited their proliferation and induced apoptosis in a time- and dose-dependent manner. Additionally, its administration reduced mitochondrial membrane potential (MMP), upregulated Bax/Bcl-2 ratio, and Cytochrome C, caspase-3, and -12 expression, showing its potential for combating lymphomas [67].

#### 4.2.2. Caspase-Independent Apoptosis

Allicin triggers caspase-independent cell death, which is followed by the release of apoptosis-inducing factor (AIF) and protein kinase A (PKA) from the mitochondria [4]. The mitochondrial protein AIF mediates apoptosis by translocating to the cytosol and the nucleus [68,69]. Previously, a study showed that allicin suppresses the proliferation of AGS cells and gastric cancer cells that express the p53 gene and trigger apoptosis through a caspase-independent mechanism, probably by activating PKA [57]. R Luo et al. [70] demonstrated that allicin prevents gastric cancer by slowing down the proliferation of cancer cells, stopping the G2/M phase of the cell cycle, reducing ER stress, and inducing mitochondria-mediated apoptosis. Additionally, an in vivo study revealed that allicin (18.2 mg/kg) efficiently protects against vestibular dysfunction caused by cisplatin by blocking both caspase-dependent and -independent apoptotic pathways [71]. According to an in vitro study, the combination of allicin (2–64 g/mL) and 5-fluorouracil (5-FU) (10–480 g/mL) significantly increased apoptosis and can reverse multidrug resistance in gastric carcinoma cells by lowering the expression of WNT5A, DKK1, MDR1, P-gp, and CD44 levels [72]. In esophageal squamous cell carcinoma, allicin (40–100 µg/mL) decreased cell viability, hindered the migration of Eca109 and EC9706 cells, and triggered G2/M phase arrest through the p53-p21-CDK1/cyclinB signaling pathway. Additionally, it inhibited the development of tumors in vivo and promoted cell apoptosis via mitochondrial signaling pathways [73]. Lastly, treatment with allicin reduced the proliferation of SGC-7901 cancer cells and caused them to undergo apoptosis. After allicin treatment, SGC-7901 cells undergo synchronous apoptosis through both intrinsic mitochondrial and extrinsic Fas/FasL-mediated mechanisms [74].

### 4.3. Role of Allicin in Sustained Proliferative Signaling

Cells have receptors that initiate intracellular signaling for cell growth and division. Cancer cells can divide without these signals, leading to uncontrolled proliferation. Mutations in the receptor’s gene maintain this growth signaling cascade, passing it onto daughter cells and potentially forming a tumor [13]. Several signaling pathways, including estrogen receptor signaling cell growth, cyclin-dependent kinases (CDKs), hypoxia-inducible factor-1 (HIF-1), NF-κBs, PI3K/Akt, and insulin-like growth factor receptor (IGF-1R), have been proposed as potential targets to inhibit cancer proliferation [48]. According to a study by Namita Pandey et al., allicin therapy (40 μg/mL) for NSCLC reduces the expression of HIF-1 and HIF-2 in hypoxic cells by inhibiting the ROS/MAPK pathway [65]. In liver cancer, allicin (40 µM ) inhibits Cholangiocarcinoma (CCA) cell proliferation and invasion through STAT3 signaling—STAT3 is a key transcription factor involved in proliferation [66]. Zelin Yang et al. indicated that a certain allicin concentration (60 μg/mL) may boost p53 expression by lowering IE2 protein levels, hence preventing the growth of glioma cells in the central nervous system [75]. In colorectal cancer, it was reported that allicin enhances X-ray radiotherapy sensitivity, possibly due to downregulating NF-κB, IKKβ mRNA, p-NF-κB, and p-IKKβ protein expression levels, both in vitro and in vivo. Also, allicin alone inhibits colorectal cancer cell proliferation by suppressing NF-κB signaling [76]. According to a study, allicin of 10 µg/mL efficiently reduces the growth and metastasis of gastric cancer cells by enhancing the expression of miR-383-5p while reducing the expressions of ERBB4, p-PI3K, and p-Akt [77]. Furthermore, a study compared the effects of allicin and cis-platinum on oral tongue squamous cell carcinoma (OTSCC) patients’ cell proliferation and apoptosis and found that allicin at a concentration of 50 µg/mL may be more effective than cis-platinum at a concentration of 40 µg/mL in treating OTSCC patients [78].

Lastly, it has been demonstrated that allicin inhibits cell proliferation and modifies several signaling pathways involved in cell growth, according to the above previous studies.

### 4.4. Role of Allicin in Evasion of Anti-Growth Signaling

The ability of cancer cells to evade anti-growth signals is an important feature. Cancer cells need to discover a way to separate themselves from the numerous signals that inhibit cell growth to continue to proliferate [79]. Cancer cell growth involves various pathways including the AT-rich interactive domain 1A (ARID1A), Hippo, growth differentiation factor 15 (GDF15), insulin-like growth factor (IGF), p53, phosphatase and tensin homolog (PTEN), retinoblastoma protein (Rb), Notch, and Krüppel-like factor 5 (KLF5) pathways [79]. In the fight against cancer, the use of tumor suppressors in gene therapy is an essential approach. Over 50% of human malignancies have a mutant form of the tumor suppressor p53 [48]. The p53 gene is a significant tumor suppressor gene that is involved in several physiological processes, including immune response, cell differentiation, stress, cell death, and the cell cycle [80]. In vitro, a study by Yung-Lin Chu et al. demonstrated that allicin is a strong active ingredient for possible adjuvant treatment in liver cancers with or without p53 deficiency or deficit [81].

Retinoblastoma tumor suppressor (RB) controls the cell-cycle transition and the response to therapy. In breast cancer, the complexes of cyclin D–cyclin-dependent kinase 4 (cyclin D–cdk4) and cyclin E–cdk2 catalyze hyperphosphorylation, inactivate RB. Consequently, it permits the progression of the cell cycle [82,83]. One study found that after 16 h of exposure to allicin (4 mg/mL dry mass), Rb in breast cancer cell line MCF7 was completely dephosphorylated [22].

Allium in garlic has a bioactive ingredient called diallyl trisulfide (DATS), which lowers the risk of cardiovascular disease, boosts immunity, and has anti-aging and anticancer properties [84]. The Notch signaling system plays a critical role in cell differentiation, proliferation, and apoptosis. It may also have a role in the carcinogenesis of osteosarcoma in vitro and in vivo. Moreover, Notch receptor expression is dysregulated in the colon, cervix, pancreas, and osteosarcoma [85]. According to research by Yonggang Li et al., DATS (50 μM) over 48 h inhibits the growth of osteosarcoma cells by downregulating the expression of Notch-1 protein and other Notch genes such Hes-1 and cyclin D1 [86].

### 4.5. Role of Allicin in Replicative Immortality

Unlimited cell division is a crucial characteristic of cancer cells, allowing them to replicate endlessly, unlike normal cells that undergo programmed cell death after a certain number of divisions. This property is achieved through genetic mutations that allow cancer cells to continue dividing even when their DNA is damaged, avoid programmed cell death, and preserve their telomeres. These telomeres undergo constant extension by telomerase (an enzyme that extends telomeres) in tumors, which helps to enhance the lifespan of the tumor cells, prevent senescence, and promote proliferation [87].

Studies have revealed that allicin could potentially inhibit replicative immortality in cancer cells through several mechanisms, including the inhibition of telomerase activity, induction of apoptosis, and regulation of proliferative signaling. Allicin could hinder the action of telomerase and trigger programmed cell death in a time-dependent and dose-dependent pattern. This effect was observed in SGC-7901 cells, which are gastric cancer cells [52]. In addition, it has been proved that allicin through the triggering of programmed cell death and controlling multiple signaling pathways involved in cell proliferation can disrupt the replicative immortality of cancer cells [12]. For instance, a study demonstrated that allicin (15.0 and 20.0 µM concentrations) exerts anticancer effects in A549 and H1299 cells by modulating the PI3K/AKT pathway. These effects include inhibition of cell proliferation, invasion, and metastasis [88,89].

### 4.6. Tumorigenesis and Carcinogen Activity Suppression

Tumorigenesis is a term that denotes the process through which a normal cell turns into tumor cells, exhibiting an uncontrolled cell division or growth, the ability to invade surrounding tissues, and epigenetic and metabolic dysregulation [90], whereas carcinogens are cancer-causing agents that can be suppressed to prevent the initiation of tumorigenesis [91]. Understanding and targeting these processes can be crucial in developing cancer treatments. One in vivo study showed that allicin effectively suppressed the tumorigenesis of colon tumors in AOM/DSS mice through a mechanism connected to the inhibition of STAT3 signaling activation [92]. Similarly, in in vitro research, DATS (diallyl trisulfide), constituents of allicin, have indicated potential to suppress carcinogenic activity in normal and cancerous breast cells via inducing apoptosis and cell cycle arrest. Additionally, they tend to repress cell proliferation and oxidative DNA damage [93].

### 4.7. Role of Allicin in Tumor-Dysregulated Metabolism

Tumor-dysregulated metabolism is the term used to describe the modified metabolic condition observed in cancer cells. Cancer cells undergo metabolic reprogramming to fulfill their heightened nutritional needs for unregulated growth. The dysregulated metabolism enables cancer cells to obtain nutrients from environments with low nutritional levels and sustain cell survival and proliferation [94]. The metabolic changes observed in cancer cells encompass enhanced glycolysis, disrupted amino acid metabolism, inhibited oxidative phosphorylation, and increased fatty acid production. These metabolic alterations are prevalent across several cancer subtypes and have a pivotal impact on the advancement of cancer and its resistance to treatment [95]. Typically, normal cells primarily generate their cellular energy by oxidative phosphorylation in the mitochondria. However, in the well-known phenomenon called the Warburg effect, cancer cells mostly produce their energy by increasing glycolysis and subsequently undergoing lactic acid fermentation. Allicin initiates the S-thioallylation of 8 out of the 10 enzymes involved in the catabolism of glucose to pyruvate in Jurkat cells. In addition, allicin also targets lactate dehydrogenase, an enzyme responsible for converting pyruvate to lactate. This enzymatic targeting mechanism results in the disruption of the capacity of the cancer cell to efficiently absorb glucose by this interference with critical glycolysis enzymes [96]. Glutathione (GSH) has a crucial role in cancer progression and chemoresistance. While it is necessary for the elimination and detoxification of carcinogens in healthy cells, greater GSH levels in malignant cells are related to tumor growth and enhanced resistance to therapy. GSH is present throughout cellular compartments at millimolar quantities and contributes to the maintenance of healthy levels of intracellular oxidative equilibrium, serving as an antioxidant and driving metabolism [97]. Allicin can easily penetrate the cell membrane and react with the cellular thiol to transiently deplete the intracellular GSH level, inducing the inhibition of cell cycle progression and growth arrest [98]. An in vitro study indicated that allicin encourages oxidative stress and autophagy in Saos-2 and U2OS (osteosarcoma cells) by modulating the MALATI-miR-376a-Wnt and β-catenin pathway [99]. In vivo investigations showed that allicin (60 mg/kg) reduces glucose and lipid levels in diabetic rats’ blood, thus helping in improving glucose tolerance [100].

### 4.8. Role of Allicin in Tumor-Promoting Inflammation

Rudolf Virchow’s observation of leukocytes within tumors in the 19th century was the initial indication of a potential association between inflammation and cancer [101]. However, in the past ten years, conclusive proof has been gathered indicating that inflammation is a crucial factor in the development of tumors, and several molecular pathways responsible for this process have been clarified [102,103]. The initial growth of a tumor is influenced by the enhancement of cell proliferation and decreased cell death, both of which are activated by pathways driven by inflammation [104]. Tumor-promoting inflammation supports the progression of cancer by establishing an appropriate environment for the proliferation of tumors and aiding in multiple phases of tumor formation and spread [105]. Inflammation-induced tumor promotion can occur at any stage of tumor development, either early or late. It can result in the activation of premalignant lesions that have remained inactive for an extended period. Inflammation can impact tumor promotion in many ways. Apart from promoting higher cell division and improved cell survival, inflammation can also trigger the activation of the angiogenic switch. This switch enables a small inactive tumor to acquire the necessary blood supply for its subsequent growth phase [104].

A major tumor-promoting mechanism is the production of tumor-promoting cytokines by immune/inflammatory cells, which stimulate premalignant cells to produce genes that promote cell proliferation and survival by activating transcription factors (NF-kB, STAT3, and AP-1) and several cytokines (IL-1, TNF, IL-6, IL-23). A study on human cytomegalovirus (HCMV)-infected glioblastoma multiforme (GBM) demonstrated that glioma cells overexpressing IE2 exhibited increased expression of IL-6 and IFN-β. Furthermore, the study found that allicin significantly inhibited the expression of these two inflammatory factors [75].

Alon Lang and his team evaluated the immunomodulatory impact of allicin on two types of intestinal epithelial cell lines, HT-29 and Caco-20. They noticed that allicin exhibited significant anti-inflammatory properties in these cells. The data suggest that allicin inhibits the production of chemokines IL-8, MIG, and IP-10, as well as the secretion of IL-1b from intestinal epithelial cells, both spontaneously and in response to TNF-a [106]. Moreover, as previously stated, allicin could hinder the proliferation and invasion of CCAs by inhibiting the STAT3 proinflammatory transcription factor mediated by SHP-1 [66]. Furthermore, a study on the Caco-2 cell line demonstrated that allicin exerts an anti-inflammatory effect by inhibiting NF-κB and suppressing the P38 and JNK pathways [107]. The PI3K/Akt/NF-κB pathway has been observed to be dose-dependently downregulated by allicin, thereby impeding the excessive synthesis of prostaglandin, NO, iNOS, and various other inflammatory mediators. Based on the studies mentioned above, it can be inferred that allicin can regulate proinflammatory pathways, thus potentially exhibiting anti-inflammatory and anticancer properties [108].

### 4.9. Role of Allicin in Angiogenesis Inhibition

Angiogenesis, which is the process of generating new blood vessels, plays a critical role in the growth and progression of the disease. It is essential for tumor growth, metastasis, and the formation of new blood vessels [109]. Angiogenesis serves as the foundation for a vast array of pathological and physiological processes, such as tumor invasion, metastasis, and primary growth. Furthermore, there is a correlation between inflammation and angiogenesis, with the expansion of blood vessels playing a critical role in developing several inflammatory-mediated diseases. Consequently, modern biology faces the challenge of inhibiting angiogenesis; developing novel antiangiogenic agents is of the utmost importance [110]. Allicin has demonstrated the ability to inhibit angiogenesis in multiple research investigations. It has been discovered that it inhibits the function of vascular endothelial cells (VECs) by reducing cell viability, migratory rate, and tube-forming ability [111]. Vascular Endothelial Growth Factor (VEGF), Fibroblast Growth Factor-2 (FGF2), Chemokines, Transforming Growth Factor-Beta (TGF-β), Platelet-Derived Growth Factor (PDGF), and Hypoxia-Inducible Factor 1-Alpha (HIF-1α) are among the essential chemical signals that regulate angiogenesis [112]. A study demonstrated that the application of allicin resulted in a considerable reduction in the expression of HIF-1α in human renal clear cell carcinoma (RCC-9863) cells. Furthermore, the downstream effectors, VEGF and Bcl-2, exhibited a reduction. In addition, the administration of allicin demonstrated a partial reversal of the effects generated by overexpression of HIF-1α, thus suggesting the protective function of allicin against cancer [113]. Allicin can also inhibit angiogenesis in lung cancer cells (A549) by reducing VEGF-A protein expression, suppressing VEGF-A gene expression, targeting the HIF pathway, and stimulating the immune system [114]. Allicin can substantially inhibit the VEGFR-2 receptors in breast cancer, according to an in silico study, which could limit the growth of breast cancer cells [115]. Furthermore, allicin effectively inhibits VEGF-C-induced lymphangiogenesis and infiltration of inflammatory cells in a Matrigel plug assay in C57BL/6 mice, suggesting its ability to limit lymphangiogenesis by relying on an inflammation-dependent mechanism [16]. AGE, one of allicin’s metabolites, can significantly impact cell behavior, motility, invasion, and angiogenesis. It has been observed that AGE can enhance the adhesion of endothelial cells to collagen and fibronectin, which can inhibit their ability to move and invade surrounding tissues. Additionally, AGE has been shown to suppress endothelial cell motility and proliferation from making tube-like structures that can develop into blood vessels [116,117]. Allicin also hinders the process of actin polymerization, which plays a role in reorganizing the cytoskeleton and is essential for forming new blood vessels (angiogenesis). Akt phosphorylation is suppressed by it. Akt, also called protein kinase B, is a pivotal kinase that plays a role in cell proliferation, survival, and angiogenesis. Phosphorylation of Akt initiates signaling pathways that enhance the growth and survival of endothelial cells, which are crucial for angiogenesis [110].

### 4.10. Role of Allicin in Tissue Invasion and Metastasis

**Figure 3:**
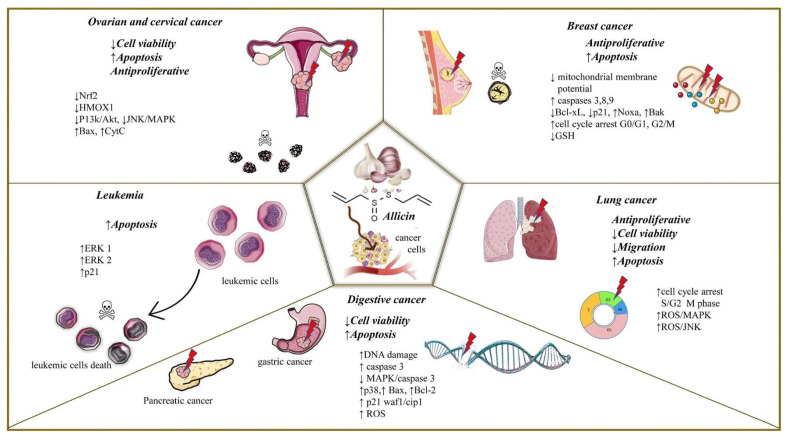
Allicin as a potent anticancer agent.

In one study, allicin was shown to have inhibitory effects on tissue invasion and metastasis in various types of cancer, including gastric carcinoma (GC); the researchers noted that allicin significantly affects the proliferation and metastasis of gastric cancer GC in both HGC27 and AGS cell lines. Treatment with allicin resulted in reduced migration and invasion abilities, indicating a lower potential for metastasis. This effect was achieved by increasing the expression of miR-383-5p, which in turn suppressed the activity of the ERBB4/PI3 K/Akt signaling pathway, which has a role in promoting cancer [77]. Another study revealed a novel finding that allicin has the potential to impede the invasion of lung adenocarcinoma cells (A549 and H1299) by modulating the balance of TIMP/MMP via suppressing the activity of the PI3K/AKT signaling pathway. This pathway is a critical initiator of intracellular signaling cascades in lung cancer, which facilitates invasion progression. Moreover, it serves as a viable therapeutic target in the development of anticancer drugs. The combination of allicin and LY294002, which is an inhibitor of PI3K, showed a significant decrease in H1299 cell invasion [89]. Allicin has demonstrated the ability to hinder the spread and secondary growth of LoVo human colon cancer cells by reducing the expression of VEGF, u-PAR, and HPA mRNA. Hence, it suggests its potential for inhibiting tumor invasion and metastasis [118]. STAT3 is involved in various cellular processes such as proliferation, survival, apoptosis, angiogenesis, and metastasis. STAT3 is a central hub for multiple cancer-causing signaling pathways through the phosphorylation of tyrosine residue 705. As previously stated, it has been demonstrated that allicin can inhibit STAT3, leading to various beneficial mechanisms in combating cancer. In a recent study, it was found that allicin has the potential to inhibit the proliferation, invasion, and metastasis of human CCA cell lines (HuCCT-1 and QBC939). The study suggests this inhibition may be achieved by targeting the SHP-1-mediated STAT3 signaling pathway [66]. Allicin is also reported to suppress the invasion and metastasis of MCF-7 cells effectively by inhibiting the activation of ERK1/2 induced by TNF-α. Based on the findings, allicin can hinder the activation of VCAM-1 caused by TNF-α. This is achieved by blocking the ERK1/2 and NF-κB signaling pathways and enhancing the interaction between ER-α and p65 [119]. Another study mentions the positive effect of allicin on tumor invasion among SiHa cells, a human cervical squamous cell carcinoma cell line, by inhibiting the PI3K/AKT pathway, which is essential for promoting cell growth and survival, and by suppressing NRF2 expression. NRF2 has been linked to the progression of cervical cancer, facilitating cell proliferation and reducing apoptosis. Aberrant activation of NRF2 in cervical cancer cells can result in inflammation and cancer formation. Allicin may potentially inhibit tumor invasion and metastasis in SiHa cells [49]. According to the study, it was discovered that AGEs could hinder the invasiveness of SW480 and SW620 cells. However, no impact was observed on the invasive activity of HT29 cells, which suggests that the anti-invasiveness of AGEs seems to rely on the specific type of cancer cell [98]. Figure 3 summarizes the mechanisms of action of allicin on different cancer types.

### 4.11. Clinical Trials: Allicin in Cancer Treatment

**Figure 4:**
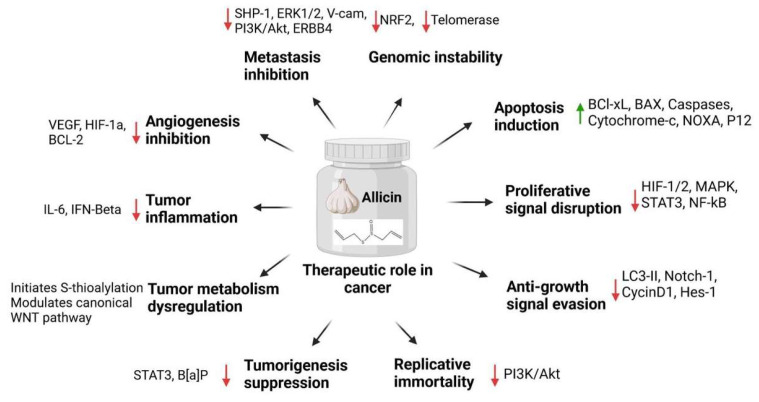
Summary of the anticancer mechanisms allicin utilizes.

Over the past few decades, numerous clinical trials have investigated the potential use of allicin in cancer treatment. In 2008, a clinical study applied allicin locally to progressive gastric carcinoma. Allicin was administered to 40 patients through gastroscopy at the lesion region 48 h before gastrectomy. The results revealed that allicin suppressed cancer cell proliferation and growth and promoted cell death. Interestingly, allicin exerted these therapeutic benefits by upsurging the FAS and BAX protein levels and decreasing the BCL2 expression in cancer tissue samples [4,98]. A randomized clinical trial was carried out in 2015 that evaluated the effectiveness of allicin on stage II submucous fibrosis, which is a precancerous health condition characterized by progressive fibrosis and inflammation. During this trial, allicin (1 mg/week) and triamcinolone acetonide (2 mg/week) were intralesionally injected into patients for 16 weeks. Allicin significantly improved the signs and symptoms associated with the disease without causing any harmful effects. This comprehensive study suggested the potential use of allicin as an adjunctive therapeutic drug [7,120]. Excessive cellular oxidative stress is widely perceived as a key factor in pathophysiological conditions and cancer development. Excessive oxidative stress is widely perceived as a regulatory factor in cancer development. Many clinical trials have been performed to assess the benefits of allicin-containing formulations/products and allicin on oxidative stress. In one trial, coated garlic powder tablets containing 900 mg of allicin (0.6%) and allin (1.3%) were given to individuals for 2 months. The outcomes demonstrated a significant increase in the level of an antioxidant, glutathione (GSH), in circulating human erythrocytes, suggesting allicin and related natural compounds as potent oxidative stress inhibitors [7]. Several preclinical trials have also investigated the protective effects of allicin against Helicobacter pylori (HP), a key factor for gastric cancer, peptic ulcers, and gastritis. However, clinical studies have supported the use of fresh garlic and its oil to improve HP infection in most gastric conditions [6]. Therapeutic applications of allicin have also been extended to treat stage III/IV follicular lymphoma (FL) patients. A non-randomized, open-label, and single-group intervention clinical study was carried out in 2008. This study sought to evaluate the anticancer effects of various dietary factors, including allicin. The therapeutic effects of the garlic-derived compound allicin on tumor cell apoptosis, proliferation, and infiltration were observed using patients’ clinical samples [121]. Moving forward, a randomized clinical trial included 20 cancer patients who were administered allicin, with 4.40 mg/g of garlic, for 21–61 days. Allicin significantly improved antioxidant status in patients undergoing chemotherapy by declining the levels of derivatives of reactive oxygen species [122]. These clinical approaches indicate that allicin exhibits potential as a robust anticancer agent, demonstrating enhanced therapeutic effectiveness while minimizing adverse effects. Figure 4 and Table 1 summarises various anticancer mechanisms utilized by allicin.

## 5. Conclusions

Natural dietary components’ availability and their potential to target many cancer hallmarks with one agent make them attractive. Garlic has been found to exhibit anticancer activities by interfering with multiple stages of carcinogenesis. Allicin not only showed a direct antitumor effect but also showed a sensitization effect toward radiotherapy and chemotherapy, as well as protecting normal cells against carcinogens. The majority of cancer medications now employ immune surveillance and anti-growth signals to stop cancer cells from proliferating uncontrollably. These pathways are essential for cancer cells to develop infinitely. For patients and doctors alike, resistance to these medications and the high expense of therapy present serious obstacles. Future research should focus on creating natural substances for treatment and prevention. Combining natural and synthetic substances can boost bioavailability or provide synergistic effects. Improved distribution of natural substances can also be achieved using novel technologies such as nanotechnology. However, there is no FDA-approved drug containing purified allicin. The data of allicin clinical trials are relatively lacking, and more patient-centered, multi-center, and large-scale studies are needed to determine the efficacy of allicin and its secondary metabolites.

## Figures and Tables

**Table 1:** Experimental design of allicin in different cancer hallmarks and the outcomes of these studies.

Cancer Hallmark	Concentration Used	Type of Cells	Experimental Model	Outcomes of the Combination	Reference
**Genomic Instability**	40 nM	Cervical cancer	vitro	Allicin inhibited the expression of NRF2 in cervical cancer SiHa cells.	[49]
	0.016 mg/mL, 0.05 mg/mL, and 0.1 mg/mL	Gastric cancer	vitro	Allicin can suppress telomerase activity and induce apoptosis of SGC-7901 cells.	[52]
**Inducing Apoptosis**	(15–120 μg/mL)	Gastric cancer	vitro	Treatment with allicin reduced the proliferation of SGC-7901 cancer cells and caused them to undergo apoptosis. After allicin treatment, SGC-7901 cells underwent synchronous apoptosis through both intrinsic mitochondrial and extrinsic Fas/FasL-mediated mechanisms.	[74]
	18.2 mg/kg	Testicular, ovarian, bladder, head and neck, lung, and cervical cancer	vivo	Allicin protected against vestibular dysfunction caused by cisplatin and potentially avoided oto-vestibulotoxicity caused by cisplatin.	[71]
	30 and 60 µg/mL	Brain cancer	vitro	Allicin can effectively inhibit proliferation and induce apoptosis of both intrinsic and extrinsic pathways in U251 glioma cells.	[63]
	allicin (10 µM) and Cisplatin (10 µM)	Thyroid cancer	vitro	The combination of allicin (10 µM) and Cisplatin (10 µM) caused autophagy-dependent cell death in SW1736 and HTh-7 cells.	[64]
	45 μM (MCF-7) and 12 μM, 20 μM, and 45 μM (HCC-70)	Breast cancer	vitro	Allicin decreased cell viability and antiproliferative effects through activation of caspase -3, -8, and -9, leading to (MCF-7 and HCC-70) cell apoptosis through upregulation of P21, NOXA, BAK, and BCL-XL.	[62]
	40 µg/mL	Lung cancer (NSCLC)	vitro	Allicin caused G2-M arrest and death in A549 cells through ROS-dependent changes to p53, p21, and other downstream effectors.	[65]
	10 and 20 mg/kg		vivo	Enhanced the apoptotic caspases by suppressing STAT3 signaling, such as cleaved caspase-3 and cleaved caspase-9	[66]
	4 μg/mL and 8 μg/mL (EL-4 cells)	Lymphoma	vitro	Increased caspase-3, -12, and Bax/Bcl2 and cytochrome-c expression and reduced mitochondrial membrane potential.	[67]
**Caspase-independent apoptosis**	40 nM	Cervical cancer	vitro	Allicin primarily inhibited NRF2 in cervical cancer cells.	[49]
	allicin (2–64 g/mL) and 5-FU (10–480 g/mL)	Gastric cancer	vitro	The combination could reverse multidrug resistance in the GC cells and lower the expression of WNT5A, DKK1, MDR1, P-gp, and CD44 levels.	[72]
	40–100 µg/mL	Oesophageal squamous cell carcinoma	vitro	Decreased cell viability and triggered G2/M phase arrest via p53-p21- CDK1/cyclinB and induced apoptosis via mitochondrial signaling pathways.	[73]
**Sustained Proliferative Signaling**	40 µg/mL	Lung cancer (NSCLC)	vitro	Allicin reduced the expression of HIF-1 and HIF-2 in hypoxic cells by inhibiting the ROS/MAPK pathway.	[65]
	40 µM	Liver cancer	vitro	Allicin inhibited CCA cell proliferation and invasion through suppressing STAT3 signaling.	[66]
	60 μg/mL	Central nervous tumor	vitro	Allicin concentration may boost p53 expression by lowering IE2 protein levels.	[75]
	10 µg/mL	Colorectal cancer	vitro	Allicin inhibited cell proliferation and migration and promoted apoptosis in HCT116 cells.	[76]
	30 mg/kg		vivo	Allicin inhibited colorectal cancer cell proliferation by suppressing NF-κB signalling.	
	10 µg/mL	Gastric cancer	vitro	Allicin efficiently reduced the growth and metastasis by enhancing the expression of miR-383-5p while reducing the expressions of ERBB4, p-PI3K, and p-Akt.	[77]
	50 μg/mL	Oral cancer	vitro	Allicin was shown to be highly effective at inhibiting cell growth and promoting cell death when compared to cis-platinum in OTSCC patients.	[78]
**Evasion of Anti-Growth Signalling**	35 µM	Liver cancer	vitro	After allicin exposure for 24 h, Hep G2 and Hep 3B cells were knocked down by p53 and exhibited inhibition of LC3-II protein expression but increased caspase-3 production.	[81]
	4 mg/mL dry mass	Breast cancer	vitro	Retinoblastoma (Rb) in breast cancer cell line MCF7 was completely dephosphorylated after 16 h of allicin exposure.	[22]
	DATS (50 μM)	Osteosarcoma cells	vitro	Diallyl trisulfide (DATS) downregulated the expression of Notch-1 protein, Hes-1, and cyclin D1.	[86]
**Replicative Immortality**	15.0 and 20.0 µM H1299 and A549	Lung adenocarcinoma	vitro	Allicin reduced cell proliferation by modulating PI3K/AKT signalling.	[89]
**Tumorigenesis and Carcinogen Activity Suppression**	AOM/DSS mice	Colon cancer	vivo	Suppressed the tumorigenesis of colon tumors by inhibition of STAT3 signalling activation.	[92]
	DATS (MCF7-10AT1 cells)	Breast cancer cells	vitro	DATS suppressed B[a]P (ubiquitous environmental pollutant) carcinogenic activity in normal and cancerous breast cells.	[93]
**Tumor Dysregulated Metabolism**	100 µM allicin (Jurkat cells)	Acute T cell leukemia	vitro	Allicin initiated the S-thioallylation of enzymes involved in the catabolism of glucose to pyruvate.	[96]
	Allicin Saos-2 and U2OS cells	Osteosarcoma	vitro	Allicin encouraged oxidative stress and autophagy in osteosarcoma cells by modulating MALATI-miR-376a-Wnt and β-catenin cascade.	[99]
	Allicin (60 mg/kg) Wistar rats (6 weeks)	Diabetes	vivo	Allicin reduced glucose and lipid levels in diabetic rats’ blood, thus improving glucose tolerance.	[100]
**Tumor- Inflammation**	Allicin 60 µg/mL U87MG cells	Human cytomegalovirus (HCMV)	vitro	Allicin significantly inhibited the expression of IL-6 and IFN-β inflammatory factors which are overexpressed in human cytomegalovirus (HCMV)-infected glioblastoma multiforme (GBM).	[75]
**Angiogenesis inhibition**	Allicin 0.1 mg/mL RCC-9863 cells	Renal carcinoma	vitro	Allicin resulted in a considerable reduction in the expression of HIF-1α in human renal clear cell carcinoma (RCC-9863) cells, also inhibiting VEGF and Bcl-2.	[113]
	50 and 100 µg/mL (A549 cells)	Lung cancer	vitro	Allicin can also inhibit angiogenesis in lung cancer cells (A549) by reducing VEGF-A protein expression, suppressing VEGF-A gene expression, targeting the HIF pathway, and stimulating the immune system.	[114]
		Breast cancer	silico	Allicin can substantially inhibit the VEGFR-2 receptors in breast cancer, according to an in silico study, which could limit the growth of breast cancer cells.	[115]
	Allicin 10 μM and 10 μg	Lymphangiogenesis	vivo	Allicin effectively inhibited VEGF-C-induced lymphangiogenesis and infiltration of inflammatory cells in a Matrigel plug assay in C57BL/6 mice.	[16]
***Tissue Invasion and Metastasis***	Allicin 10 µg/mL	Gastric cancer	vitro	Allicin increased the expression of miR-383-5p, which in turn suppressed the activity of the ERBB4/PI3 K/Akt signalling pathway, which has a role in promoting cancer.	[77]
	Allicin 7.5 and 10.0 µM	Lung adenocarcinoma	vitro	Allicin has the potential to impede the invasion of lung adenocarcinoma cells (A549 and H1299) by modulating the balance of TIMP/MMP via suppressing the activity of the PI3K/AKT signalling pathway.	[89]
	Allicin 3 and 6 µg/mL	Colon cancer	vitro	Allicin has demonstrated the ability to hinder the spread and secondary growth of LoVo human colon cancer cells by reducing the expression of VEGF, u-PAR, and HPA mRNA.	[118]
	Allicin 20 µM	Cholangiocarcinoma	vitro	Allicin has the potential to inhibit the proliferation, invasion, and metastasis of human CCA cell lines (HuCCT-1 and QBC939). The study suggests this inhibition may be achieved by targeting the SHP-1-mediated STAT3 signalling pathway.	[66]
	Allicin 0.1–10 ng/mL	Breast cancer	vitro	Allicin suppressed the invasion and metastasis of MCF-7 cells effectively by inhibiting the activation of ERK1/2 induced by TNF-α. Based on the findings, allicin can hinder the activation of VCAM-1 caused by TNF-α. This is achieved by blocking the ERK1/2 and NF-κB signalling pathways and enhancing the interaction between ER-α and p65.	[119]
	Allicin 20 nM	Cervical cancer	vitro	Allicin may potentially inhibit tumor invasion and metastasis in SiHa cells by inhibiting the PI3K/AKT pathway, which is essential for promoting cell growth and survival, and by suppressing NRF2 expression. NRF2 has been linked to the progression of cervical cancer.	[49]
	0, 0.1, 1, and 10 mg/mL	Colorectal cancer	vitro	AGEs could hinder the invasiveness of SW480 and SW620 cells by enhancing the adhesion of endothelial cells to collagen and fibronectin, which can inhibit their ability to move and invade surrounding tissues.	[98]

## Data Availability

Data are available within the article.
